# Role of a mental health physician in the management of oncology patients: a case vignette and the need for collaboration

**DOI:** 10.3389/frhs.2024.1385370

**Published:** 2024-05-02

**Authors:** Aishatu Yusha'u Armiya'u, Yusuf Akande

**Affiliations:** ^1^ Department of Psychiatry, Federal University of Health Sciences Azare, Bauchi, Nigeria; ^2^ Department of Psychiatry, College of Medical Sciences, Abubakar Tafawa Balewa University, Bauchi, Nigeria; ^3^ Department of Clinical Services, Federal Neuropsychiatric Hospital, Kaduna, Nigeria

**Keywords:** cancer, mental health, PACER, oncology, culture, collaborative care

## Abstract

There is an interplay between oncology and mental health, resulting in a high prevalence of mental disorders among cancer patients. Out of the several interventions developed to target cancer specifics, collaborative care is indicated due to its efficacy. The perspective delves into the efficacy of collaborative care models, spotlighting a culturally informed strategy designed to harmonize mental and physical health interventions to bolster the overall wellbeing and resilience of individuals battling cancer. Central to our discussion is a compelling case vignette of Raliat, a patient diagnosed with ovarian cancer whose narrative exemplifies the multifaceted challenges cancer patients face, including stigma, psychological distress, and social isolation. Raliat's story illuminates the profound impact of cultural beliefs on patient experiences and the critical importance of a sensitive, holistic approach to care that respects cultural contexts. Through this lens, our analysis reveals that addressing emotional and situational stressors through collaborative care can significantly reduce oxidative stress, potentially decelerating the progression of both cancer and accompanying mental health disorders. We advocate for integrating mental health services into oncological care, drawing on the case vignette to argue for policies that facilitate such merger by employing validated collaborative care models. We conclude with a call for public education to diminish cancer stigma and improve social outcomes, emphasizing the use of a culture-informed PACER (physical, affective, cognitive, environmental, and relationship) strategy in providing comprehensive care for cancer patients and their families.

## Introduction

A complex bi-directional relationship exists between mental health conditions and cancers, where each domain significantly influences the other, creating a complex interplay of biological, psychological, and social factors. Recent advances in the neurobiology of cancer pinpoint this interconnection, with Mravec providing a comprehensive definition and historical overview, along with outlining the clinical implications of the neurobiological pathways involved in cancer progression and its impact on mental health (1). This body of work highlights the pivotal role of the nervous system in the development and spread of cancer and the modulation of the tumor microenvironment, which, in turn, influences psychological wellbeing.

On the other hand, the work of Dhabhar, “Effects of stress on immune function: the good, the bad, and the beautiful,” highlights the effects of stress on immune function, illustrating how acute stress can enhance immune responses, while chronic stress can lead to immunosuppression (2). This dynamic is particularly relevant in the context of cancer, where stress-related immune modulation can affect tumor growth and metastasis and the psychological resilience of patients.

Multiple pathways have been suggested to explain this comorbidity including psychoneuroimmunology. One of which is the free radical theory. Free radicals are unstable molecules that are naturally produced in the body as byproducts of the breakdown of nutrients from bodily processes or exposure to toxic substances (residual waste). The production of free radicals at a pace that exceeds the neutralization capacity of antioxidants causes oxidative stress, which has been implicated in cancer, depression, and high anxiety levels. Free radical-induced chromosomal defects and oncogene activation are associated with the initiation and progression of cancer (3, 4). Inflammation and immunological suppression have been linked to long-term stress (physical or psychosocial) (5). In addition, stress contributes to cardiovascular and neurodegenerative illnesses, as shown by the strong correlation between cancer and traumatic life experiences (6). Workplace stress was identified as a risk factor for a number of cancers in a meta-analysis that included 281,290 individuals (7).

Within a socio-ecological framework, the bi-directional relationship between mental health conditions and cancers is further complicated by individual, community, and systemic interactions. At the individual level, genetic predispositions, lifestyle factors, and psychological resilience play critical roles. Community factors, including social support networks and cultural attitudes toward cancer and mental health, significantly influence patient outcomes. Systemically, healthcare access, policy frameworks, and the integration of mental healthcare in oncology settings are crucial for addressing the complex needs of cancer patients.

By situating the discussion within this framework, we can better understand the multifaceted interactions at play, emphasizing the need for a holistic approach to oncology care. This approach acknowledges the convergence of neurobiological insights with psychosocial and environmental factors, advocating for integrated care strategies that address the full spectrum of patient needs. In doing so, it becomes imperative to leverage recent scientific insights and adopt a comprehensive perspective that spans from molecular mechanisms to societal influences, thereby ensuring that cancer patients receive care that is not only medically effective but also psychosocially supportive.

## Cancer impact on mental health

Cancer is known to impact mental health and wellbeing. Moderate to high anxiety scores were reported by approximately 40% of cancer survivors, while moderate to high depression scores were reported by approximately 20%, with non-grief-related depression possibly related to inflammation or the effects of treatment and grief related to functional losses (8). Cancer-related cognitive impairment (CRCI) is known to include changes in memory, executive function, attention, and processing speed. CRCI occurs in about 30% of patients prior to treatment and up to 75% during treatment (9).

The neurotoxicity of chronic stress from cancer and cancer treatment has been reported (10). Also, chemotherapy or radiation can result in loss of hearing and tinnitus (11). A correlation between hearing loss, depression, and cognitive decline has been reported (12), with lower cognitive performance among cancer survivors occurring up to 20 years post-treatment (13).

## Psychosocial morbidity in cancer

A recent review of psychiatric disorders among cancer patients in sub-Saharan Africa reported a high incidence (14), with 66.9% presenting with a Diagnostic Statistical Manual-IV disorder (15). Major depression and anxiety were most prevalent, with diagnostic surveys revealing 16.4%–40.3% of patients were experiencing depression and 8.8%–19.0% experiencing anxiety (16–19). Screening tools indicated even higher rates (20–23). In addition, opioid use, dysthymia, post-traumatic stress disorder (PTSD), and other psychiatric conditions were noted (15, 24). Contributing factors included being single, lack of social support, and advanced cancer (19, 25), which also increased the risk of suicidality and poorer psychosocial outcomes (26). In a study in the United States, authors emphasized the need for the establishment of culturally appropriate tools and programs aimed at lessening the mental health burden among cancer patients (27). Furthermore, they suggested formulating policies that would facilitate the merging of mental healthcare into standard cancer treatment by utilizing validated models like the collaborative care approach (27).

In Nigeria, the psychological toll of cancer is substantial, with 75% of patients experiencing psychological distress (28). Studies reveal a 27.5% prevalence of major depressive disorder among cancer patients, much higher than the 9.5% in the general population (19). Patients often turn to spirituality and structured problem-solving strategies to cope with the challenges posed by their diagnosis, highlighting the critical need for integrated mental health services in oncological care (29, 30).

## Need for collaborative care

Various forms of cancer-specific interventions have been developed to target the conditions resulting from the emergence of psychological disorders and maladjustment to cancer. This includes different formats of psychotherapy (individual, group, and family therapy) and orientations (psychodynamic, supportive-expressive, cognitive-behavioral, existential, and meaning-centered). However, the choice of intervention is informed by the clinical and psychological condition, type and phase of illness, context, and availability of psycho-oncology services (multidisciplinary teams) (31). Collaboration was given priority in the research on the effectiveness of targeted interventions since it has been shown to improve quality of life and overall wellbeing by lessening the severity of physical and mental symptoms (32). However, an urgent need for a systematic approach was pinpointed (31).

## Target of collaborative care

**Figure 1:**
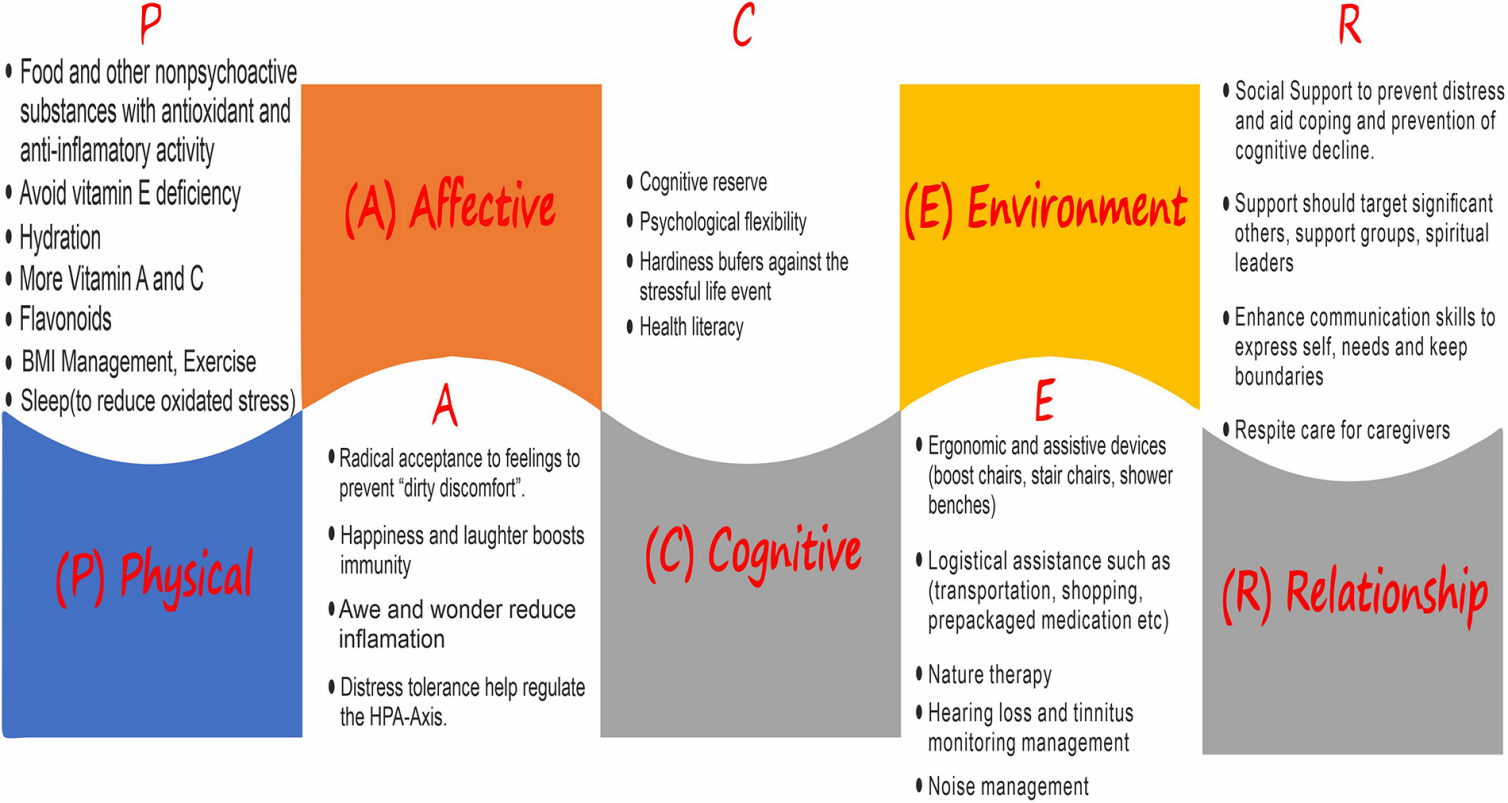
PACER strategy.

Collaborative care should target the physical, affective, cognitive, environmental, and relationship (PACER) aspects (Figure 1). This could be carried out both proactively and reactively (33); however, we opine it should be informed by culture. Physical relates to sourcing for antioxidants and anti-inflammatory activity in food and other non-psychoactive substances, sleep, exercise, and hydration, among others. The affective deals with facing reality rather than denial through radical acceptance of feelings; trying to stay happy and still have reasons to laugh helps in reducing pain while increasing endorphins and boosting immunity. Cognitive entails cognitive reserve, psychological flexibility (mindful awareness and making a decision of best use of one's energy), and hardiness. Importantly, health literacy informs people and aids their understanding of how physical, affective, cognitive, environmental, and relationship aspects could help. Environment tackles ergonomics and logistical assistance such as transportation. Social support, enhanced communication skills, and respite care are central to the relationship component.

## A case scenario

Raliat (real name withheld), a marketer in her late 30s, was diagnosed with ovarian cancer. She felt her life was about to come to an end and did not see reasons why she should do anything with or for anyone else. Considering she has not been on good terms with her mother-in-law, she was sure her diagnosis was a curse from her mother-in-law. She found comfort in getting drunk often as it temporarily helped her to forget her worries. Raliat hoped she died of cancer soon enough, or else she would have to end it all herself.

Although Raliat's husband could not help but wonder how his wife got cancer, the reality dawned on him that his wife was no longer interested in having sex. He dreaded life as a widower and felt ashamed of seeing his wife drunk. Raliat's children wondered what the fate of their mother would be, although friends at school had already told them their mother would die, and since cancer is hereditary, they would also have the diagnosis in a couple of years. Friends and neighbors mostly ignored them, while some laughed at them. What hurt them the most was their mother's irrational acts when drunk at night, which neighbors and passersby found amusing.

Neighbors believed that ovarian cancer, in particular, is a highly communicable consequence of adultery. They were resolute on isolating Raliat and excluded her from the community women's group and other social gatherings. Women in the community also warned their children and wards to stay away from Raliat's children and residence.

**Figure 2:**
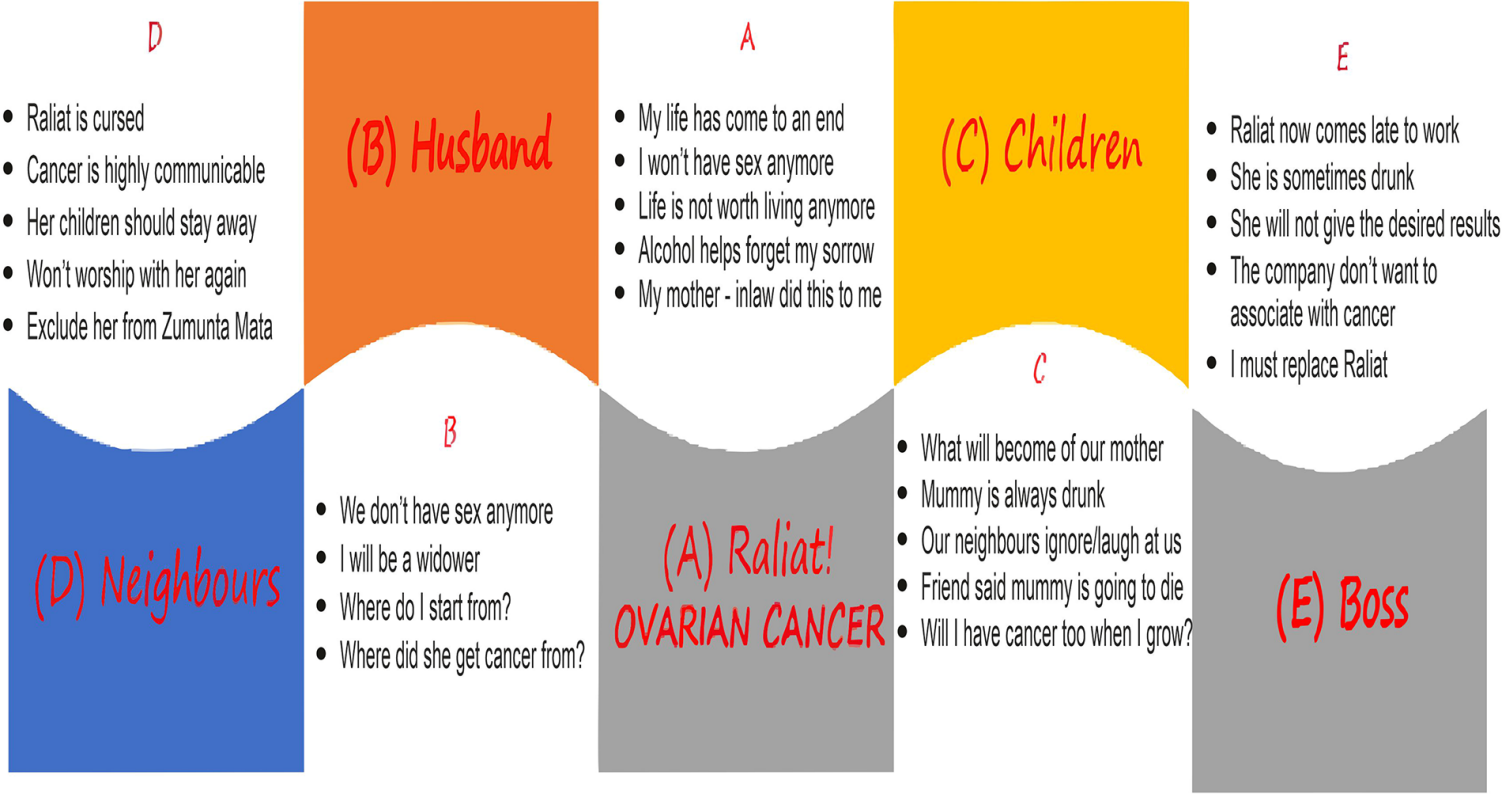
Cancer diagnosis of Raliat.

At work, the director observed that Raliat was not as prompt and timely as before. He was informed of Raliat's diagnosis and did not feel that should justify her lateness to work and lesser productivity. Rather, he sensed that a diagnosis of cancer among staff would send a wrong message to existing and prospective partners, hence the need to replace Raliat immediately. The scenario is illustrated in Figure 2.

Raliat's journey from the despair of an ovarian cancer diagnosis to finding hope and support emphasizes the necessity of a culturally informed PACER strategy in the management of cancer patients. This approach holistically addresses her needs, taking into account the cultural context of her experience.

### Interventions

Physical (P): Raliat was introduced to a tailored physical wellness program designed to mitigate the side effects of cancer treatment and enhance her overall wellbeing. This included nutritional guidance focused on local, culturally appropriate foods known for their anti-inflammatory and antioxidant properties, and a gentle exercise regimen aligned with her physical capacity and cultural preferences.
Affective (A): To address her emotional wellbeing, Raliat was provided with psycho-oncology support that included culturally sensitive counseling and therapy sessions. These interventions aimed at validating her feelings, reducing her emotional distress, and leveraging culturally relevant forms of expression, such as storytelling and music, to help her process her diagnosis and treatment journey.
Cognitive (C): Cognitive interventions focused on educating Raliat about her disease and treatment options in a culturally resonant manner. This included discussions on the importance of mental health in cancer care and training in cognitive-behavioral techniques (CBTs) to help her challenge negative thoughts and foster a positive, resilient mindset.
Environmental (E): Recognizing the impact of Raliat's surroundings on her recovery, efforts were made to engage her community and workplace in her care. Community outreach programs educated her neighbors and colleagues about cancer, addressing myths and reducing stigma. Environmental modifications, such as creating a comfortable and soothing space at home and negotiating flexible working arrangements, were also implemented.
Relationship (R): To bolster Raliat's social support network, interventions included family counseling sessions to improve communication and understanding among her relatives and connect her with cancer support groups. These groups were carefully selected to ensure cultural compatibility, providing Raliat with a sense of community and belonging.

### Outcomes

Physical and emotional wellbeing: Raliat experienced significant improvements in her physical health, reporting reduced treatment side effects and increased energy levels. Emotionally, she felt more balanced and hopeful, attributing this to the comprehensive support and understanding she received.
Enhanced knowledge and coping skills: Through cognitive and educational interventions, Raliat gained a deeper understanding of her condition and became adept at employing coping strategies to navigate her emotional response to cancer.
Community reintegration: The environmental and relationship interventions successfully mitigated Raliat's social isolation. She reconnected with her community, which now showed greater empathy and support, reducing the stigma she initially faced.
Strengthened family bonds: Family counseling sessions improved communication and empathy within Raliat's family, creating a stronger support system that empowered her throughout her treatment journey.
Sustained engagement with care: Empowered by a network of support and a comprehensive care plan, Raliat remained actively engaged in her treatment, demonstrating a high level of adherence and participation in decision-making processes.

## Discussion

Raliat's case, characterized by a culturally informed PACER strategy, echoes findings from similar studies that emphasize the importance of holistic and integrated care approaches in oncology. Studies such as those by Kotronoulas et al. highlight the positive impacts of psycho-oncological interventions on patient wellbeing, similar to Raliat's improved mental and physical health outcomes (34). While Raliat's story illustrates the potential benefits of a PACER-based approach, its generalizability to other cultural contexts is limited. The effectiveness of certain interventions, particularly those involving community engagement and the use of culturally specific coping strategies, may not translate directly to settings with different cultural norms and healthcare systems.

### Social ecological framework exploration

At an individual level, personal beliefs, genetic predispositions, and individual health behaviors play a critical role. Raliat's case emphasizes the importance of addressing personal coping mechanisms and psychological resilience in oncology care. Family dynamics, social support networks, and patient–provider relationships are key at the interpersonal level. In Raliat's scenario, family counseling and community education interventions were vital, reflecting findings that strong social support enhances patient outcomes.

At an organizational level, healthcare systems and oncology care settings influence the delivery and efficacy of interventions. The multidisciplinary approach in Raliat's case illustrates the benefit of cohesive care coordination within healthcare organizations. Cultural norms, stigma associated with cancer and mental health, and community support systems are influential at the community level. Raliat's engagement with her community and the educational outreach performed are examples of strategies to combat stigma and enhance communal support.

Policy level is representative of the healthcare policies, access to care, and national mental health strategies that shape the broader context within which care is delivered. The successful integration of mental health services into oncology care, as seen in Raliat's case, requires supportive policies and funding mechanisms.

### A glance at Nigeria

In Nigeria, the development of a collaborative oncology care model is evolving (35), particularly with efforts focused on enhancing inter-professional collaboration and building capacity for conducting oncology clinical trials (36). A significant initiative in this regard involves partnerships with several Nigerian institutions aimed at strengthening local research capabilities and improving facilities for conducting advanced oncology clinical trials. This model is underpinned by active engagement with various stakeholders, policymakers, and international collaborations that support and train local investigators (36).

However, the implementation of such models faces barriers, including the current low rate of inter-professional collaborative practice in healthcare settings. A study that assessed inter-professional collaborative practice at the tertiary care level in Nigeria found that health professionals rated the practice as low and perceived it as negatively impacting patient outcomes, professional performance, job satisfaction, and the frequency of conflicts and strike actions (35). Also, infrastructural limitations, insufficient maintenance of medical equipment, and a skills gap among local investigators and clinical trial staff were challenges highlighted during assessments of oncology centers, revealing deficiencies in areas such as laboratory and imaging capabilities, oncology nursing, chemotherapy facilities, and data management (36).

Possible models for collaborative care in Nigeria could involve policy formulation and commitment from healthcare professionals to embrace teamwork and patient-centered care. Such models would necessitate integrating various healthcare providers, including mental health specialists, into oncology care settings to ensure a culturally attuned holistic approach to patient care.

## Conclusion

Several mental health conditions result from the diagnosis and treatment of cancer, necessitating collaborative efforts that involve mental health physicians. The implementation of the PACER (physical, affective, cognitive, environmental, relationship) collaborative care model within oncology represents a pivotal shift toward a more holistic and integrated approach to cancer care. This model not only acknowledges but actively incorporates the myriad of factors influencing patient outcomes into the care process, ranging from the biological to the psychosocial, and is grounded within a cultural context. By doing so, it sets forth a comprehensive framework that significantly enhances the traditional paradigms of oncology care.

## Implications

In practice, the PACER model mandates a broadened scope of patient assessment, transcending physical symptoms to include psychological and social wellbeing, thereby fostering an informed understanding of patient needs. It expresses the importance of interdisciplinary collaboration by involving a mental health physician in a diverse team of healthcare professionals to offer a cohesive and personalized treatment plan. Furthermore, it places a strong emphasis on cultural competency, urging providers to tailor care strategies to respect and incorporate patients’ cultural backgrounds and preferences.

From a policy perspective, the PACER model calls for robust support for integrated care models that blend mental and physical health services seamlessly. It advocates for policies that incentivize collaborative practices and cultural sensitivity in healthcare delivery, emphasizing the need for resources and training that enable the implementation of such models. These policy changes are pivotal in creating a healthcare environment that supports the comprehensive care approach the PACER model represents.

Future research directions are clearly delineated by the implementation of the PACER model. Studies focusing on the effectiveness of PACER across diverse cultural and clinical settings are crucial for validating and refining the model. Longitudinal research on patient outcomes can provide deeper insights into the long-term benefits of collaborative care, while investigations into barriers to implementation can guide strategies for wider adoption.

The novel aspects of the PACER approach, including its multidimensional focus and emphasis on cultural sensitivity, introduce significant innovations to oncology care. By addressing the full spectrum of factors affecting cancer patients, the PACER model advocates for a transformative change in patient care, promising not only to enhance patient experiences and outcomes but also to set a new standard for holistic, patient-centered oncology care. Its successful implementation could mark a significant advancement in the field, leading to a paradigm shift in how cancer care is conceptualized and delivered.

## Data Availability

The original contributions presented in the study are included in the article/Supplementary Material, further inquiries can be directed to the corresponding author.
